# Association of maternal hemoglobin levels and chorioamnionitis with preterm birth and low birth weight: a cohort study

**DOI:** 10.1007/s12519-026-01041-6

**Published:** 2026-05-29

**Authors:** Wen Yu, Yan-Hui Hao, Si-Yue Chen, Jia-Ying Wu, Si-Wei Zhang, Chen Zhang, Yan-Ting Wu, He-Feng Huang

**Affiliations:** 1 Shanghai Key Lab of Reproduction and Development, Institute of Reproduction and Development, Obstetrics and Gynecology Hospital, Fudan University, No. 419 Fangxie Rd, Shanghai 200011, China; 2 Shanghai Key Lab of Female Reproductive Endocrine Related Diseases, Shanghai, China

**Keywords:** Anemia, Birth weight, Birth outcomes, Chorioamnionitis, Low birth weight, Maternal hemoglobin, Preterm birth

## Abstract

**Background:**

Approximately one-quarter of infants worldwide are born either preterm, with a low birth weight, or both. Maternal anemia significantly increases the risk of adverse birth outcomes. We aimed to investigate the association between hemoglobin levels—alone and in conjunction with chorioamnionitis—and birth outcomes.

**Methods:**

This retrospective cohort study included 54,300 pregnant women who delivered between January 2018 and December 2023 and whose antenatal examination records were available at a university-affiliated hospital in Shanghai, China. The primary outcomes were preterm birth and low birth weight.

**Results:**

In our cohort, 33.9% of mothers were diagnosed with anemia. A low hemoglobin concentration (hemoglobin concentration < 100 g/L) during the third trimester was significantly associated with an increased risk of preterm birth [adjusted odds ratio (aOR) = 1.87; 95% confidence interval (CI) = 1.56–2.24] and low birth weight (aOR = 1.45; 95% CI = 1.20–1.76). Notably, the co-occurrence of moderate to severe anemia and chorioamnionitis was associated with higher odds of preterm birth and low birth weight in the third trimester, with aORs (95% CI) of 3.26 (1.92–5.52) and 3.02 (1.78–5.11), respectively.

**Conclusions:**

Maternal anemia, particularly during the third trimester, is associated with an increased risk of preterm birth and low birth weight. The co-occurrence of anemia and chorioamnionitis further amplifies these risks.

**Graphical abstract:**

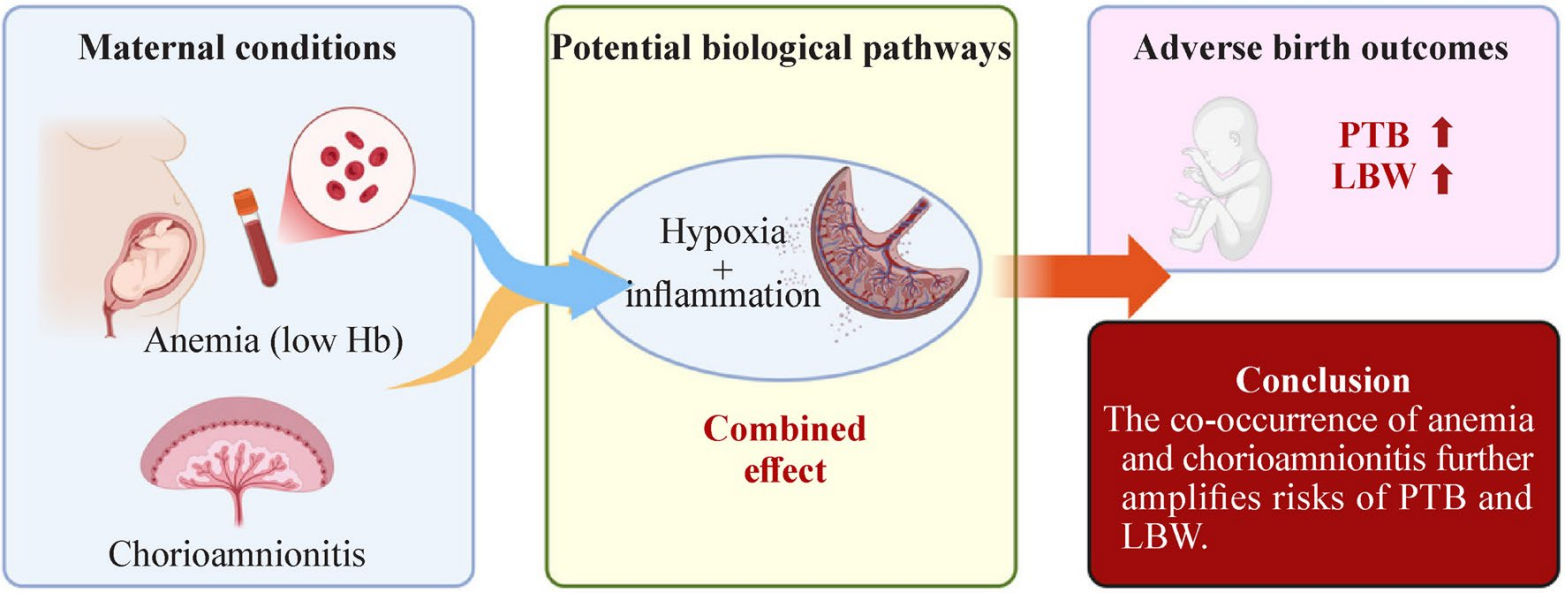

**Supplementary Information:**

The online version contains supplementary material available at 10.1007/s12519-026-01041-6.

## Introduction

Anemia is a common complication during pregnancy and is characterized by an insufficient number of red blood cells or reduced oxygen-carrying capacity, as defined by the World Health Organization (WHO) [1]. Clinical symptoms include fatigue, weakness, lethargy, irritability, and decreased work tolerance [2]. Globally, the prevalence of anemia among pregnant women is approximately 36.5%, exceeding that of the non-pregnant population [3]. China’s Hospital Quality Monitoring System indicates a 17.78% prevalence of anemia among pregnant women aged 15–49 years [4]. A study conducted in Shanghai in 2021 reported an even higher prevalence of maternal anemia (30.2%), underscoring its significant health impact [5]. Anemia is a major global risk factor for spontaneous preterm birth (PTB) and small-for-gestational-age (SGA) infants [6]. In accordance with the updated 2016 WHO guidelines, anemia during pregnancy is defined as a hemoglobin (Hb) concentration < 110 g/L in the first and third trimesters or < 105 g/L in the second trimester [7]. Maternal anemia is a potentially modifiable risk factor during pregnancy, and emerging evidence suggests that timely identification and optimal treatment are associated with improved perinatal outcomes, underscoring the clinical importance of monitoring and managing Hb levels throughout gestation [8].

Chorioamnionitis refers to inflammation of the amniochorionic membrane and is a significant cause of maternal and neonatal morbidity [9]. It affects 1%–6% of pregnancies in the United States, although data on its prevalence in Asian populations remain limited [10]. Chorioamnionitis is commonly caused by ascending infections and is characterized histologically by increased neutrophil infiltration, necrosis, thickening of the amnion basement membrane, and chorionic microabscesses [9, 11]. Associated complications include increased maternal intensive care unit admission, blood transfusions, uterine rupture, and unplanned surgical interventions following delivery [12, 13]. It is frequently associated with premature rupture of membranes [14]. Infections can disrupt fetal growth trajectories, leading to infants who are small because of intrauterine growth restriction or PTB [15, 16].

The combined impact of maternal Hb levels and chorioamnionitis on birth outcomes has not been extensively examined in birth cohorts. PTB and low birth weight (LBW) are critical indicators of neonatal health and are associated with long-term risks, including neurodevelopmental disorders, as well as respiratory and gastrointestinal complications [17]. This study aimed to investigate the association between maternal Hb levels—both independently and in combination with chorioamnionitis—and adverse birth outcomes.

## Methods

### Study design and participants

This retrospective cohort study included all singleton births delivered between January 1, 2018, and December 31, 2023, at the Obstetrics and Gynecology Hospital of Fudan University, a tertiary care center in Shanghai, China. We extracted data on maternity delivery records, Hb levels, related test reports, and birth outcomes from the electronic medical system. The inclusion criteria were as follows: (1) availability of obstetric prenatal records and (2) receipt of prenatal care and delivery at the study hospital. The exclusion criteria included (1) missing maternal demographic or pregnancy outcome data; (2) a maternal history of severe liver or kidney dysfunction, cancer, or autoimmune system disease; and (3) missing infant sex or birth weight data.

This study used retrospective and fully de-identified data extracted from the hospital information system. As such, no direct contact was made with the participants, and no identifiable personal information was used or disclosed. The study was reviewed and approved by the Ethics Committee of the Obstetrics and Gynecology Hospital of Fudan University (approval number: 2021-90). The requirement for informed consent was formally waived by the ethics committee in accordance with institutional and national guidelines for retrospective studies involving anonymized data. This research was also registered in the Chinese Clinical Trial Registry (ChiCTR2100047245).

### Data collection, measurements, and outcomes

Anemia during pregnancy was defined according to the WHO guidelines: Hb concentration < 110 g/L in the first (≤ 14 weeks) and third trimesters (≥ 28 weeks) and < 105 g/L in the second trimester. Hb levels were also defined as “high” if they exceeded 130 g/L [18, 19]. Hb levels were evaluated separately in the first, second, and third trimesters according to trimester-specific cutoffs. When multiple Hb measurements were available within the same trimester, the first recorded Hb value in that trimester was used for analysis.

Obstetric complications and birth outcomes were obtained from electronic medical records and included gestational diabetes mellitus (GDM), gestational hypertension disease (GHD), preeclampsia, postpartum hemorrhage, and PTB (< 37 gestational weeks, both spontaneous and medically indicated), LBW (< 2500 g), macrosomia (> 4000 g), SGA, large-for-gestational-age (LGA), fetal distress, and birth defects. LGA was defined as a birth weight > 90th percentile for gestational age-specific birth weight distribution [20]. Similarly, SGA was defined as a birth weight < the 10th percentile for gestational age [21].

Chorioamnionitis diagnosis was based on hospital records using either clinical or histological criteria, including maternal fever (≥ 37.8 °C) accompanied by two or more of the following signs: maternal tachycardia (> 100 beats/min), fetal tachycardia (> 160 beats/min), uterine tenderness, purulent or foul-smelling amniotic fluid or vaginal discharge, and maternal leukocytosis (> 15,000/mm^3^) [10]. The primary predictors were maternal Hb levels, which were evaluated as both continuous and categorical variables.

### Statistical analysis

Descriptive statistics were used to summarize the baseline characteristics of the mother‒child dyads. Continuous variables and categorical variables are described as the mean (standard deviation) and number (percentage), respectively. To examine the association between maternal Hb levels and adverse birth outcomes, Hb levels were categorized into four groups: moderate to severe anemia (Hb < 100 g/L), mild anemia (Hb 100–109 g/L), normal (Hb 110–129 g/L), and high Hb (Hb ≥ 130 g/L). The primary outcomes were PTB and LBW.

Univariate and multivariate logistic regression models were used to estimate the odds ratios (ORs) and 95% confidence intervals (CIs) for PTB and LBW associated with each Hb category, with the normal Hb group used as the reference. To examine the combined association of chorioamnionitis and maternal anemia with adverse birth outcomes, we fitted multivariable logistic regression models using a four-level joint exposure variable. We additionally included a product term between anemia and chorioamnionitis to explore potential interactions on the odds ratio scale and performed stratified analyses by chorioamnionitis status. Hb levels and chorioamnionitis were classified as binary variables.

All the models were adjusted for potential confounders of birth outcomes and determined a priori on the basis of previous knowledge. Potential confounding variables included local Shanghai residence status, maternal age, pre-pregnancy body mass index (BMI), parity, GDM, maternal education level and neonatal sex. All the statistical tests were two-sided, and a *P* value < 0.05 was considered to indicate statistical significance. Statistical analyses were performed using R version 4.2.3, with the packages mgcv, rms, ggplot2, and visreg.

## Results

### Characteristics of participants

A total of 54,300 singleton pregnant women were eligible for analysis. Of these, 5856 met the exclusion criteria, resulting in a cohort of 48,444 pregnant women included in the final analysis (Supplementary Fig. 1). The basic characteristics of the study population are presented in Table 1; 47,669 (98.4%) participants were of Han ethnicity (self-reported), and 37,711 (77.8%) pregnancies were nulliparous. The mean maternal age was 31.7 ± 4.0 years, and the preconception BMI was 21.8 ± 3.1 kg/m^2^.

**Table 1:** Basic characteristics of the participants (*N* = 48,444)

Characteristics	*n* (%)
Maternal characteristics	
Age (y), mean (SD)	31.7 (4.0)
Pre-pregnancy BMI (kg/m^2^), mean (SD)	21.8 (3.1)
Ethnicity	
Han	47,669 (98.4)
Other	775 (1.6)
Shanghai local residence	24,535 (50.6)
Parity	
Nulliparous	37,711 (77.8)
Multiparous	10,733 (22.2)
Education	
High school and lower	13,930 (28.8)
Undergraduate	23,499 (48.5)
Graduate and higher	11,015 (22.7)
Mode of delivery	
Vaginal	30,147 (62.2)
Cesarean	18,297 (37.8)
Preterm birth	2096 (4.3)
GDM	7426 (15.3)
Gestational hypertension	3528 (7.3)
Preeclampsia	3078 (6.4)
Postpartum hemorrhage	1048 (2.2)
Chorioamnionitis	2209 (4.6)
*Mycoplasma genitalium* infection	464 (1.0)
Anemia in pregnancy	16,406 (33.9)
Morbidity of anemia	
First trimester	1147 (3.0)
Second trimester	4005 (11.5)
Third trimester	14,828 (31.7)
Neonatal characteristics	
Male	25,225 (52.1)
Female	23,219 (47.9)
Gestational age (wk), median (IQR)	40 (39–40)
Birth length (cm), mean (SD)	49.7 (1.6)
Birth weight (g), mean (SD)	3294 (465)
LBW	1978 (4.1)
Macrosomia	2509 (5.2)
SGA	4639 (9.6)
LGA	3137 (6.5)

*BMI* body mass index (calculated as weight in kilograms divided by height in meters squared), *GDM* gestational diabetes mellitus, *LBW* low birth weight, *SGA* small for gestational age, *LGA* large for gestational age, *SD* standard deviation, *IQR* interquartile range

The prevalence of anemia during pregnancy was approximately 33.9%. On the basis of the diagnostic criteria for anemia in different trimesters, the number of cases and incidence rates of anemia in the first, second, and third trimesters were 1147 (3.0%), 4005 (11.5%), and 14,828 (31.7%), respectively. The mean Hb levels in each trimester are presented in Supplementary Table 1. Consistent with the curve-fitting depicted in Supplementary Fig. 2, the overall trend indicated a decrease in maternal Hb levels during pregnancy.

The prevalence rates of GDM, GHD, preeclampsia, postpartum hemorrhage, and chorioamnionitis were approximately 15.3% (*n* = 7426), 7.3% (*n* = 3528), 6.4% (*n* = 3078), 2.2% (*n* = 1048), and 4.6% (*n* = 2209), respectively. The prevalence rates of adverse birth outcomes, including PTB, LBW, macrosomia, SGA, and LGA, were approximately 4.3% (*n* = 2096), 4.1% (*n* = 1978), 5.2% (*n* = 2509), 9.6% (*n* = 4639), and 6.5% (*n* = 3137), respectively.

### Associations between hemoglobin levels and the risk of preterm birth

As displayed in Tables 2, 3 and 4 and Supplementary Fig. 3, both high Hb levels and low Hb levels were significantly associated with gestational age in the second and third trimesters. In the first trimester, Hb concentrations of 100–109 g/L significantly affected the risk of PTB (OR = 1.45; 95% CI = 1.07–1.96) (Table 2), although Hb levels in the first trimester had a limited effect on gestational age. Starting from the second trimester, moderate to severe anemia was associated with an increased risk of PTB (Table 3). In the third trimester, we found that mild anemia also increased the risk of PTB (OR = 1.39; 95% CI = 1.25–1.55) (Table 4). Additionally, Hb concentrations ≥ 130 g/L increased the risk of PTB during the second trimester (OR = 1.55; 95% CI = 1.27–1.88) (Table 3).

**Table 2:** Analysis of birth outcomes in pregnant individuals with hemoglobin levels in the first trimester (*N* = 38,303)

Variables	< 100 (*n* = 242)	100–109 (*n* = 905)	110–129 (*n* = 2133)	≥ 130 (*n* = 15,824)
Preterm birth				
*n* (%)	13 (5.3)	48 (5.3)	814 (3.8)	698 (4.4)
aOR (95% CI)	1.32 (0.75–2.32)	**1.45 (1.07**–**1.96)**	Reference	1.07 (0.96–1.18)
LBW				
*n* (%)	14 (5.7)	37 (4.0)	782 (3.6)	640 (4.0)
aOR (95% CI)	1.56 (0.91–2.70)	1.13 (0.80–1.59)	Reference	1.06 (0.95–1.18)
Macrosomia				
*n* (%)	5 (2.0)	37 (4.0)	1021 (4.7)	873 (5.5)
aOR (95% CI)	0.39 (0.16–0.94)	0.87 (0.62–1.22)	Reference	1.02 (0.93–1.12)
SGA				
*n* (%)	30 (12.3)	81 (8.9)	2033 (9.5)	1440 (9.1)
aOR (95% CI)	1.42 (0.96–2.10)	0.93 (0.74–1.18)	Reference	0.93 (0.23–1.06)
LGA				
*n* (%)	8 (3.3)	48 (5.3)	1286 (6.0)	1068 (6.7)
aOR (95% CI)	0.49 (0.24–1.00)	0.88 (0.65–1.18)	Reference	1.00 (0.92–1.09)

Data were adjusted for Shanghai local residence status, maternal age, pre-pregnancy body mass index, parity, gestational diabetes mellitus status, maternal education level and neonatal sex. Bold font indicates significant results for multivariate analysis. *LBW* low birth weight, *SGA* small for gestational age, *LGA* large for gestational age, *aOR* adjusted odds ratio, *CI* confidence interval

**Table 3:** Analysis of birth outcomes in pregnant individuals with hemoglobin levels in the second trimester (*N* = 34,747)

Variables	< 95 (*n* = 518)	95–104 (*n* = 3487)	105–129 (*n* = 28,960)	≥ 130 (*n* = 1782)
Preterm birth				
*n* (%)	48 (9.2)	151 (4.3)	1169 (4.0)	123 (6.9)
aOR (95% CI)	**2.49 (1.84**–**3.38)**	1.14 (0.95–1.35)	Reference	**1.55 (1.27**–**1.88)**
LBW				
*n* (%)	33 (6.3)	134 (3.8)	1099 (3.7)	138 (7.7)
aOR (95% CI)	**1.74 (1.22**–**2.50)**	1.03 (0.86–1.24)	Reference	**1.99 (1.65**–**2.40)**
Macrosomia				
*n* (%)	38 (7.3)	173 (4.9)	1452 (5.0)	83 (4.6)
aOR (95% CI)	**1.61 (1.15**–**2.26)**	1.10 (0.94–1.30)	Reference	**0.78 (0.62**–**0.99)**
SGA				
*n* (%)	31 (7.1)	257 (7.3)	2727 (9.4)	245 (13.7)
aOR (95% CI)	0.74 (0.53–1.03)	**0.74 (0.65**–**0.84)**	Reference	**1.62 (1.41**–**1.87)**
LGA				
*n* (%)	44 (8.4)	242 (6.9)	1774 (6.1)	124 (6.9)
aOR (95% CI)	**1.47 (1.07**–**2.02)**	**1.25 (1.09**–**1.44)**	Reference	0.98 (0.81–1.18)

Data were adjusted for Shanghai local residence status, maternal age, pre-pregnancy body mass index, parity, gestational diabetes mellitus status, maternal education level and neonatal sex. Bold font indicates significant results for multivariate analysis. *LBW* low birth weight, *SGA* small for gestational age, *LGA* large for gestational age, *aOR* adjusted odds ratio, *CI* confidence interval

**Table 4:** Analysis of birth outcomes among pregnant individuals with hemoglobin levels in the third trimester (*N* = 46,829)

Variables	< 100 (*n* = 2576)	100–109 (*n* = 12,252)	110–129 (*n* = 28,448)	≥ 130 (*n* = 3553)
Preterm birth				
*n* (%)	150 (5.8)	553 (4.5)	972 (3.4)	142 (3.9)
aOR (95% CI)	**1.87 (1.56**–**2.24)**	**1.39 (1.25**–**1.55)**	Reference	1.09 (0.91–1.31)
LBW				
*n* (%)	124 (4.8)	464 (3.7)	997 (3.5)	175 (4.9)
aOR (95% CI)	**1.45 (1.20**–**1.76)**	1.12 (1.00–1.25)	Reference	**1.36 (1.15**–**1.60)**
Macrosomia				
*n* (%)	194 (7.5)	713 (5.8)	1388 (4.8)	162 (4.5)
aOR (95% CI)	**1.71 (1.46**–**2.01)**	**1.22 (1.11**–**1.34)**	Reference	0.88 (0.74–1.04)
SGA				
*n* (%)	168 (6.5)	940 (7.6)	2897 (10.1)	460 (12.9)
aOR (95% CI)	**0.62 (0.53**–**0.73)**	**0.74 (0.69**–**0.80)**	Reference	**1.31 (1.18**–**1.46)**
LGA				
*n* (%)	251 (9.7)	917 (7.4)	1690 (5.9)	205 (5.7)
aOR (95% CI)	**1.76 (1.53**–**2.03)**	**1.28 (1.17**–**1.39)**	Reference	0.93 (0.80–1.08)

Data were adjusted for Shanghai local residence status, maternal age, pre-pregnancy body mass index, parity, gestational diabetes mellitus status, maternal education level and neonatal sex. Bold font indicates significant results for multivariate analysis. *LBW* low birth weight, *SGA* small for gestational age, *LGA* large for gestational age, *aOR* adjusted odds ratio, *CI* confidence interval

### Associations between hemoglobin levels and the risk of low birth weight

The associations between maternal Hb levels during each trimester and birth weight are illustrated in Supplementary Fig. 4. Lower maternal Hb levels were linked to lower birth weight, particularly in the second and third trimesters. As shown in Tables 3 and 4, moderate to severe anemia was associated with an increased risk of LBW from the second trimester to delivery. Moreover, we found that Hb concentrations ≥ 130 g/L also increased the risk of LBW in both the second and third trimesters.

We further analyzed the correlation between Hb concentrations ≥ 130 g/L and pregnancy complications, including GHD, GDM, and preeclampsia. Hb levels ≥ 130 g/L were significantly associated with an increased risk of GDM, whereas no statistically significant associations were observed for GHD or preeclampsia (Supplementary Table 2). As GDM is a well-established risk factor for adverse birth outcomes, it was considered a potential confounder in subsequent analyses.

### Combined association of chorioamnionitis and anemia with PTB and LBW during the third trimester

Additionally, we reported that anemia during the third trimester increased the risk of chorioamnionitis (Supplementary Table 3). The risk of PTB and LBW increased with decreasing Hb levels in mothers with chorioamnionitis, particularly the risk of LBW, compared with mothers without chorioamnionitis (Supplementary Figs. 5 and 6).

Table 5 presents the combined and infection-stratified analyses of maternal anemia and chorioamnionitis in relation to PTB and LBW to clarify their independent and combined associations and whether the association between anemia and these outcomes differed by chorioamnionitis status. Moderate to severe anemia was associated with PTB (adjusted OR = 1.84; 95% CI = 1.54–2.20) and LBW (adjusted OR = 1.43; 95% CI = 1.18–1.73). Although chorioamnionitis was associated with an increasing trend toward PTB and LBW, the association was not statistically significant. Pregnant women with both anemia and chorioamnionitis had a 3.26-fold increased risk of PTB (95% CI = 1.92–5.52) and a 3.02-fold increased risk of LBW (95% CI = 1.78–5.11). Stratified analysis revealed that mothers with both anemia and chorioamnionitis had a 3.52-fold increased risk of PTB (95% CI = 1.69–7.36) and a 2.80-fold increased risk of LBW (95% CI = 1.36–5.77).

**Table 5:** Individual and combined associations of anemia and chorioamnionitis with the risk of PTB and LBW during the third trimester

Characteristics	*N*	Preterm		LBW	
		*n* (%)	aOR (95% CI)	*n* (%)	aOR (95% CI)
Hb category^a^					
Normal	28,448	972 (3.4)	1.00 (reference)	997 (3.5)	1.00 (reference)
Moderate to severe anemia	2576	150 (5.8)	**1.84 (1.54**–**2.20)**	124 (4.8)	**1.43 (1.18**–**1.73)**
Chorioamnionitis^b^					
No	44,690	1732 (3.9)	1.00 (reference)	1671 (3.7)	1.00 (reference)
Yes	2139	85 (4.0)	**1.24 (1.02**–**1.50)**	89 (4.2)	**1.33 (1.10**–**1.61)**
Combined association^c^					
No chorioamnionitis					
Normal	27,239	931 (3.4)	1.00 (reference)	954 (3.5)	1.00 (reference)
Moderate to severe anemia	2452	138 (5.6)	**1.56 (1.32**–**1.85)**	112 (4.6)	**1.25 (1.04**–**1.50)**
With chorioamnionitis					
Normal	1209	41 (3.4)	0.96 (0.70–1.32)	43 (3.6)	0.99 (0.72–1.35)
Moderate to severe anemia	124	12 (9.7)	**3.26 (1.92**–**5.52)**	12 (9.7)	**3.02 (1.78**–**5.11)**
Stratified by infection^c^					
No chorioamnionitis					
Normal	27,239	931 (3.4)	1.00 (reference)	954 (3.5)	1.00 (reference)
Moderate to severe anemia	2452	138 (5.6)	**1.78 (1.47**–**2.14)**	112 (4.6)	**1.35 (1.11**–**1.66)**
With chorioamnionitis					
Normal	1209	41 (3.4)	1.00 (reference)	43 (3.6)	1.00 (reference)
Moderate to severe anemia	124	12 (9.7)	**3.52 (1.69**–**7.36)**	12 (9.7)	**2.80 (1.36**–**5.77)**

Bold font indicates significant results for multivariate analysis. *LBW* low birth weight, *PTB* preterm birth, *Hb* hemoglobin, *GDM* gestational diabetes mellitus, *aOR* adjusted odds ratio, *CI* confidence interval. ^a^Anemia was defined as a hemoglobin concentration below the normal range at least once in the third trimester. In the individual analysis of Hb categories, the reference group was the normal Hb group. Models were adjusted for chorioamnionitis, Shanghai local residence, maternal age, pre-pregnancy body mass index, parity, GDM, maternal education level and neonatal sex; ^b^for the analysis of chorioamnionitis, the no chorioamnionitis group was used as the reference group. Models were adjusted for Hb level, Shanghai local residence, maternal age, pre-pregnancy body mass index, parity, GDM, maternal education level and neonatal sex; ^c^for the combined association analysis, the reference group was normal Hb without chorioamnionitis. For the stratified analysis, the reference group was the normal Hb group within each chorioamnionitis stratum. Models were adjusted for Shanghai local residence status, maternal age, pre-pregnancy body mass index, parity, GDM, maternal education level and neonatal sex

## Discussion

Globally, the prevalence of anemia among pregnant women aged 15–49 years decreased from 41% (95% CI = 39–43) in 2000 to 36% (34–39) in 2019. The prevalence of anemia in 2019 varied by region, with West and Central Africa having the highest prevalence (52%) and high-income countries having the lowest prevalence (15%) [22]. Our study population comprised a cohort from Shanghai, which is a developed region, where the incidence of maternal anemia during pregnancy reached 33.9%, and the prevalence of chorioamnionitis was 4.6%. In addition to infections, nutritional deficiencies are a primary contributor to anemia [23]. In Shanghai, despite good sanitary conditions and high economic levels, the current incidence of anemia remains substantially elevated, which may reflect gaps in preconception care and health education.

Newborns who are preterm or have LBW account for the majority of neonatal deaths worldwide [16]. This study examined the associations between maternal Hb levels and PTB and LBW, which are linked to lifelong health challenges. Our findings revealed that maternal anemia, particularly during the third trimester, is significantly associated with increased risks of PTB and LBW. We also reported that compared with healthy pregnant women, pregnant women with moderate to severe anemia in the third trimester had 3.26-fold and 3.02-fold increased risks of PTB and LBW, respectively, when anemia was combined with chorioamnionitis.

The association between anemia and PTB was evident, with the risk of PTB increasing as Hb levels decreased [24–26]. This association was particularly strong in the second and third trimesters, where moderate to severe anemia significantly increased the risk of PTB. These findings are consistent with those of previous studies that reported increased odds of PTB with lower Hb levels across different trimesters [27]. Physiological stress and compromised oxygen-carrying capacity in anemic mothers likely contribute to PTB, emphasizing the importance of maintaining adequate Hb levels throughout pregnancy [28, 29].

With respect to birth weight, maternal anemia has been associated with low-birth-weight neonates [30–32]. Similarly, this study revealed that lower Hb levels were linked to lower birth weights, particularly in the second and third trimesters. The increased risk of LBW with decreasing Hb levels aligns with the findings of previous studies that identified anemia as a significant risk factor for suboptimal fetal growth [33, 34]. Reduced oxygen delivery to the fetus in anemic pregnancies may hinder fetal development, leading to LBW [35]. These findings reinforce that the third trimester is a critical period for fetal weight gain and that anemia during this stage is associated with poor birth outcomes, underscoring the need for early detection and treatment of anemia to ensure adequate fetal growth and development.

In addition to low Hb levels, high Hb levels have been considered a risk factor, given the U-shaped relationship between Hb levels and adverse birth outcomes [36]. In our study, Hb levels were stratified into four groups—moderate to severe anemia, mild anemia, normal, and high Hb—across three trimesters. We found that Hb concentrations ≥ 130 g/L increased the risk of PTB and LBW in the second and third trimesters but had a minimal effect in the first trimester. Approximately 5.1% of the included population had Hb concentrations ≥ 130 g/L during the second trimester, and this percentage increased to 7.6% during the third trimester. As early as 1986, Murphy et al. proposed that the occurrence of perinatal death, LBW, and PTB was greater in women with elevated Hb levels than in those with intermediate Hb levels during pregnancy [37]. Recent studies have also indicated that in the third trimester, maternal Hb levels are associated with birth weight in an inverted U-shaped curve and with the risks of LBW and SGA in extended U-shaped curves, including a study based on Chinese populations [30, 31, 38]. The mechanisms underlying the increased risk of adverse birth outcomes associated with high Hb levels remain unclear but may be associated with pregnancy-induced hypertension [36, 39]. Interestingly, the risk of developing GHD, GDM, and preeclampsia was significantly greater in the Hb ≥ 130 g/L group than in the anemic or normal groups. Therefore, high Hb levels also require careful monitoring, especially in the second and third trimesters, as they may be closely associated with GHD, GDM, and preeclampsia.

Chorioamnionitis, a syndrome characterized by maternal and fetal signs of both local and systemic inflammation, affects 1%–6% of term gestations [40]. Importantly, the combined association analysis revealed that women with both moderate to severe anemia and chorioamnionitis had the highest odds of having PTB and LBW during the third trimester. Reproductive tract infections—caused by viruses, bacteria, or other pathogens—have become major public health issues because of their high and increasing prevalence [41]. These infections have a profound impact on fertility and infant survival [42]. Among them, chorioamnionitis is considered the most significant infection associated with PTB and is estimated to occur in 40%–80% of preterm deliveries [17, 43]. Pathogenesis research has indicated that inflammatory mediators during chorioamnionitis, including microbial products and cytokines such as interleukin-6, tumor necrosis factor α, matrix metalloproteinase 8, and alarmins, gain access to the maternal compartment and induce a local inflammatory response, resulting in preterm labor or premature rupture [40, 44]. Pregnant women with both anemia and chorioamnionitis had a markedly higher risk of these adverse outcomes than those with either condition alone did. This synergistic effect highlights the compounded risks faced by pregnant women with concurrent anemia and infections, suggesting that integrated management strategies targeting both conditions might improve maternal and neonatal outcomes. Therefore, we propose that pregnant women with anemia during late pregnancy should not only correct anemia but also take preventive measures against chorioamnionitis.

The study findings underscore the importance of screening for and addressing anemia before and during pregnancy. Given the significant associations between low Hb levels and adverse birth outcomes, healthcare providers should prioritize interventions to maintain optimal Hb levels, especially in the second and third trimesters. Furthermore, identifying and effectively managing infections—especially chorioamnionitis—in anemic pregnant women may help further reduce the risk of PTB and LBW.

These clinical observations may be explained by the underlying biological mechanisms. From a mechanistic perspective, maternal anemia and chorioamnionitis may contribute to adverse birth outcomes through complementary biological pathways. Maternal anemia can impair oxygen delivery and placental function, leading to fetal hypoxia and restricted growth [29, 32]. Moreover, chorioamnionitis represents a state of intrauterine inflammation characterized by the activation of pro-inflammatory cytokines, which can trigger uterine contractions and premature rupture of membranes [17, 40]. Increasing evidence suggests that PTB is closely linked to dysregulated maternal–fetal immune responses, in which excessive or premature inflammation disrupts immune tolerance during pregnancy [45]. Therefore, the co-occurrence of anemia and chorioamnionitis may exert a synergistic effect by combining hypoxic stress and inflammatory activation, thereby increasing the risk of PTB and LBW.

Although this study provides valuable insights, it has several limitations. First, the retrospective design may be affected by incomplete data, and the findings may not be generalizable to populations outside the study setting. Future research should focus on prospective studies to validate these findings and further explore the underlying biological mechanisms linking anemia and chorioamnionitis with adverse birth outcomes. Second, PTB was analyzed as an overall outcome without further subclassification (e.g., < 34 vs. ≥ 34 weeks or spontaneous PTB). Although such stratification may provide additional clinical insights, maternal Hb was already categorized in detail by trimester and severity, and further subdivision would have resulted in small numbers of events in multiple subgroups, thereby reducing statistical power and compromising the stability of the estimates. Therefore, the findings should be interpreted as associations with overall PTB risk. Finally, pregnancy-related comorbidities such as GDM and hypertensive disorders may influence perinatal outcomes. Although we adjusted for GDM, residual confounding from these conditions cannot be fully excluded. Future prospective studies with more detailed phenotyping are warranted to validate these findings and further explore the underlying mechanisms linking maternal anemia and chorioamnionitis with adverse birth outcomes.

In conclusion, this study highlights the critical relationship between maternal Hb levels and the risks of PTB and LBW, with a particular emphasis on the compounded risks associated with concurrent chorioamnionitis. Ensuring optimal maternal Hb levels and effectively managing infections during pregnancy are essential strategies for improving both maternal and neonatal health outcomes. These findings highlight the need for increased clinical awareness and proactive management of anemia and infections among pregnant women to reduce the incidence of PTB and LBW.

## Supplementary Information

Below is the link to the electronic supplementary material.

Supplementary file 1 (PDF 451 KB)

## Data Availability

A deidentified analytic dataset will be available from the corresponding author upon reasonable request, beginning 1 year after publication of this article. Requests for data should be accompanied by a research proposal and evidence of ethical approval from the investigator’s institution, and will be subject to review by the principal investigators of the participating centers.
