# Supplementary material for: Association of maternal hemoglobin levels and chorioamnionitis with preterm birth and low birth weight: a cohort study
Source: World J Pediatr. 2026 May 29;22(5):511–21. doi: 10.1007/s12519-026-01041-6 (PMC13290792; doi:10.1007/s12519-026-01041-6)
Supplement: Supplementary file 1 — (PDF 451 KB) [file 12519_2026_1041_MOESM1_ESM.pdf]

Supplementary Table 1. Hemoglobin levels during different trimesters

Hb in different trimester	*n*	Min	Max	Mean	SD
1st trimester	38,303	59	168	127.57	9.26
2nd trimester	34,747	62	154	115.28	9.04
3rd trimester	46,829	62	161	115.18	10.41

SD standard deviation

Supplementary Table 2. Associations between hemoglobin levels and significant pregnancy complications

Variables	GHD		GDM		Pre-eclampsia	
	n **(%)**	**aOR (95% CI)**	***n*** **(%)**	**aOR (95% CI)**	***n*** **(%)**	**aOR (95% CI)**
Moderate to severe anemia	191 (6.5)	1.01 (0.94–1.09)	329 (11.1)	**0.60 (0.53–0.68)**	197 (6.7)	1.09 (0.94–1.27)
Mild anemia	912 (6.8)	0.95 (0.88–1.03)	1628 (12.1)	**0.67 (0.63–0.72)**	784 (5.8)	0.94 (0.86–1.02)
Normal	2333 (7.4)	1.00 (reference)	5317 (16.9)	1.00 (reference)	2018 (6.4)	1.00 (reference)
High Hb	92 (15.5)	**2.10 (1.67–2.64)**	152 (25.7)	**1.67 (1.38–2.02)**	79 (13.3)	**2.06 (1.62–2.63)**

Adjusted for Shanghai local status, maternal age, parity, and maternal education level. Bold font indicates significant results for multivariate analysis. *GHD* gestational hypertension disease, *GDM* gestational diabetes mellitus, *Hb* hemoglobin, *aOR* adjusted odds ratio, *CI* confidence interval

Supplementary Table 3. Analysis of chorioamnionitis among pregnant women with hemoglobin levels during the three trimesters

Trimester	Hb level (g/L)	*N*	Chorioamnionitis	
			n **(%)**	**aOR (95% CI)**
1st trimester	< 100	242	9 (3.7)	0.87 (0.44–1.70)
	100 to ≤ 109	905	32 (3.5)	0.85 (0.59–1.22)
	110 to ≤ 129	21,332	932 (4.3)	1 (reference)
	≥ 130	15,824	774 (4.8)	1.05 (0.95–1.16)

2nd trimester	< 95	518	34 (6.5)	**1.65 (1.16–2.36)**
	95 to ≤ 104	3487	141 (4.9)	**1.19 (1.01–1.41)**
	105 to ≤ 129	28,960	1269 (4.3)	1 (reference)
	≥ 130	1782	74 (4.1)	0.84 (0.66–1.08)
3rd trimester	< 100	2576	124 (4.8)	**1.25 (1.03–1.51)**
	100 to ≤ 109	12,252	664 (5.4)	**1.34 (1.21–1.47)**
	110 to ≤ 129	28,448	1209 (4.2)	1 (reference)
	≥ 130	3553	142 (3.9)	0.89 (0.75–1.06)

Adjusted for Shanghai local residence, maternal age, pre-pregnancy body mass index, parity, maternal education level, and neonatal sex. Bold font indicates significant results for multivariate analysis. *Hb* hemoglobin. *aOR* adjusted odds ratio, *CI* confidence interval

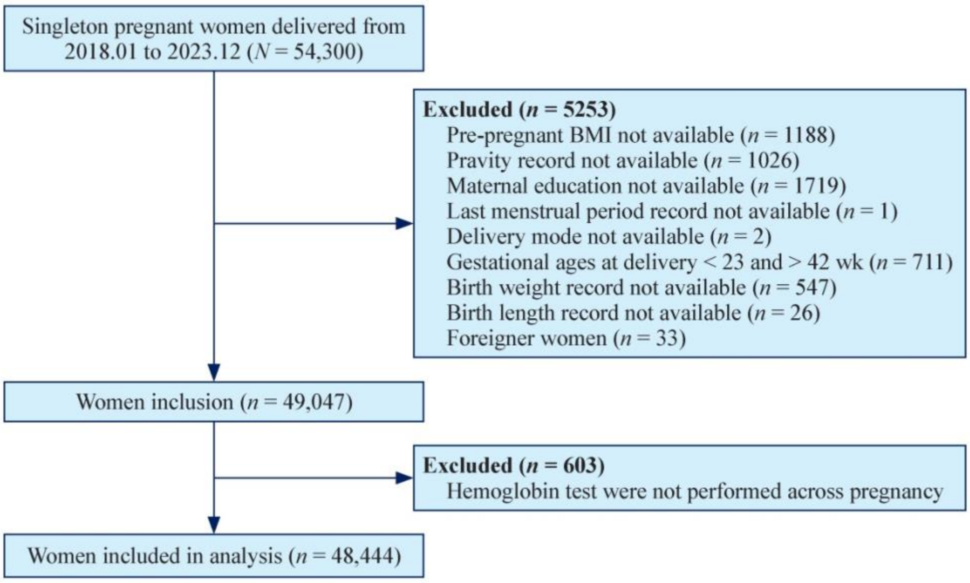

Supplementary Fig. 1 Flow chart of participants included in the study. *BMI* body mass index

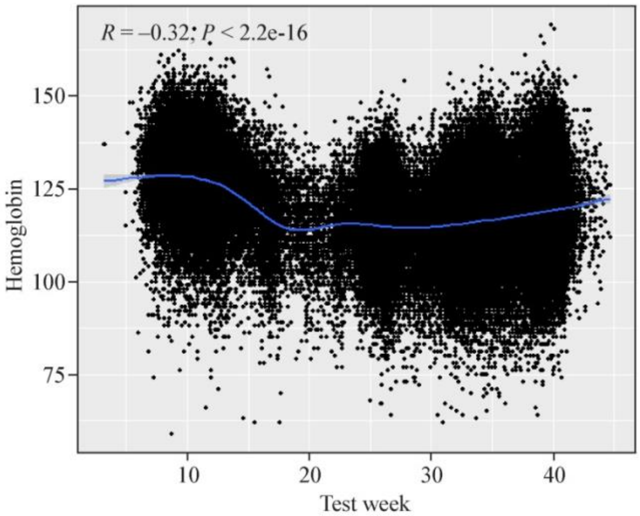

Supplementary Fig. 2 Curve-fitting of hemoglobin levels during pregnancy. Scatter plot and fitted curve of hemoglobin concentration at different stages of pregnancy in the study population

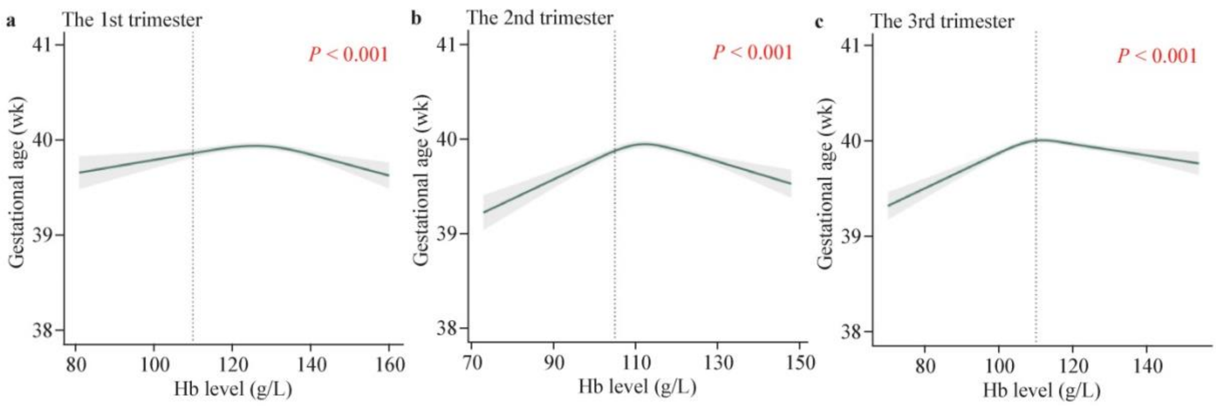

Supplementary Fig. 3 Association between maternal hemoglobin (Hb) levels and gestational age. The graphs show the associations between Hb levels and gestational age in the 1st trimester (**a**), 2nd trimester (**b**), and 3rd trimester (**c**). Data are expressed as the estimated mean gestational age with 95% confidence intervals (shaded areas), adjusted for Shanghai local residence, maternal age, pre-pregnancy body mass index, parity, gestational diabetes mellitus status, maternal education level and neonatal sex

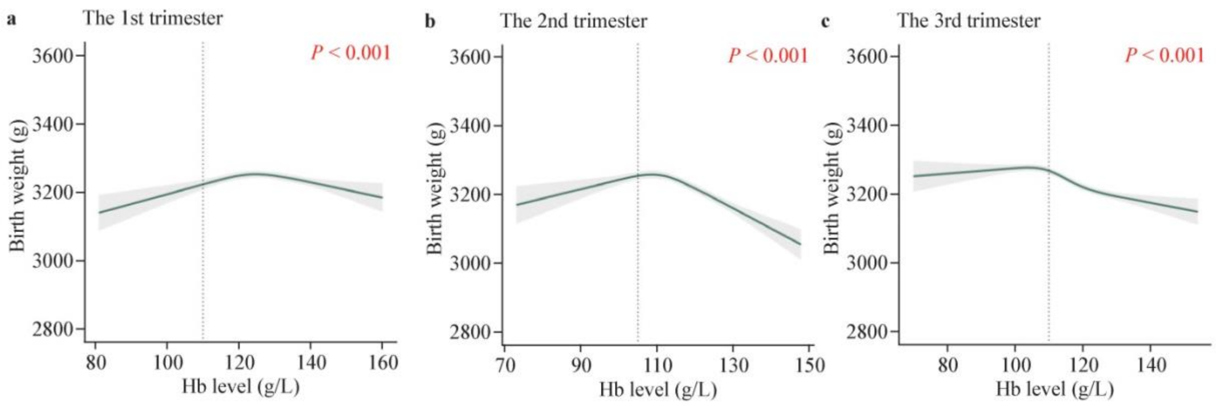

Supplementary Fig. 4 Association between maternal hemoglobin (Hb) levels and birth weight. The graphs show the associations between Hb levels and birth weight in the 1st trimester (**a**), 2nd trimester (**b**), and 3rd trimester (**c**). Data are expressed as the estimated mean birth weight with 95% confidence intervals (shaded areas), adjusted for Shanghai local residence, maternal age, pre-pregnancy body mass index, parity, gestational diabetes mellitus status, maternal education level, and neonatal sex

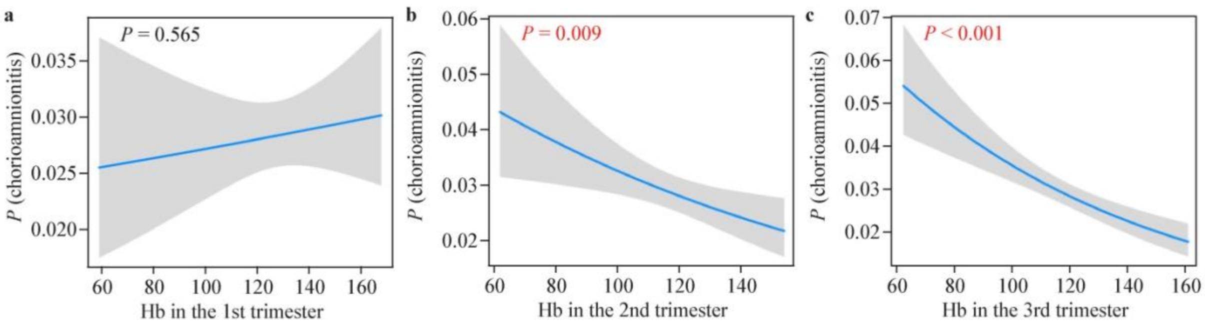

Supplementary Fig. 5 The impact of hemoglobin (Hb) levels during different trimesters on chorioamnionitis. Associations of the risk of chorioamnionitis with Hb levels during the 1st trimester (**a**), 2nd trimester (**b**), and 3rd trimester (**c**). Data are expressed as the estimated mean risk with 95% confidence intervals (shaded areas), adjusted for Shanghai local residence status, maternal age, pre-pregnancy body mass index, parity, maternal education level and neonatal sex

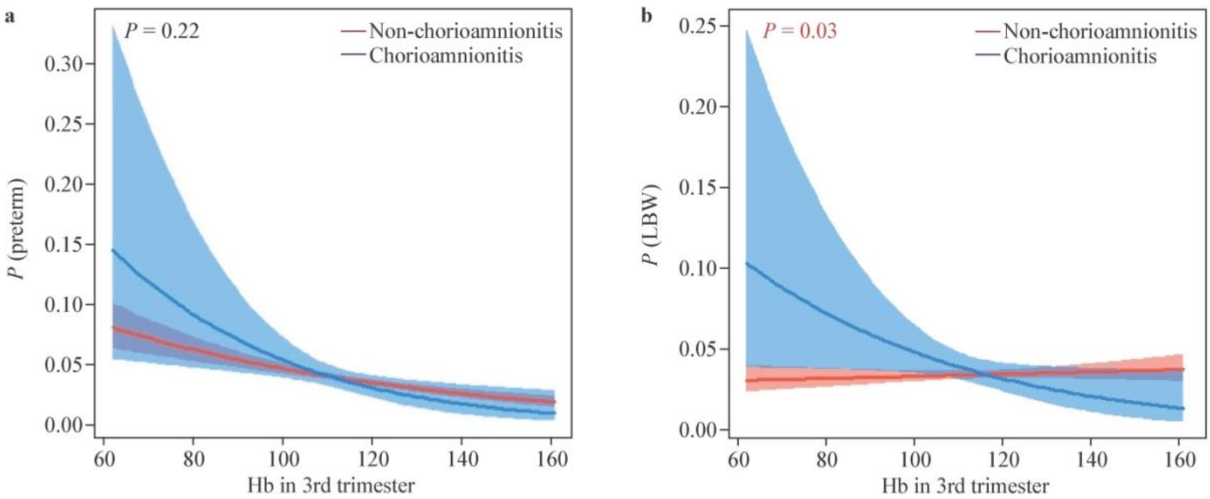

Supplementary Fig. 6 Additive associations between maternal hemoglobin (Hb) levels and chorioamnionitis with the risk of preterm birth and low birth weight (LBW) during the 3rd trimester. **a** Association of risk of preterm birth with Hb levels during the 3rd trimester stratified by chorioamnionitis; **b** association of risk of LBW with Hb levels during the 3rd trimester stratified by chorioamnionitis. Data are expressed as the estimated mean risk with 95% confidence intervals (shaded areas), adjusted for Shanghai local residence, maternal age, pre-pregnancy body mass index, parity, maternal education level, and neonatal sex
